# A Review of the Dose Justification of Phase 3 Trials to Regulatory Authorities for Drugs Intended for the Treatment of Type 2 Diabetes in Europe

**DOI:** 10.3389/fphar.2021.626766

**Published:** 2021-04-28

**Authors:** Jeroen V. Koomen, Jasper Stevens, Margje H. Monster-Simons, Hiddo J. L. Heerspink, Peter G. M. Mol

**Affiliations:** ^1^ Department of Clinical Pharmacy and Pharmacology, University of Groningen, University Medical Center Groningen, Groningen, Netherlands; ^2^ Dutch Medicines Evaluation Board (CBG-MEB), Utrecht, Netherlands

**Keywords:** Type 2 diabetes, Dose-finding, dose-response relationship, Dose selection, regulatory evaluation, cardiovascular outcome trials

## Abstract

**Aims:** Cardiovascular outcome trials with anti-diabetic drugs suggest that additional cardiovascular benefit can be achieved independent of improving glycaemic control. Nonetheless, dose selection of anti-diabetic drugs is typically based solely on glycaemic effects. We evaluated whether off-target drug effects are currently considered for dose justification to regulatory agencies.

**Methods:** In the European Union, anti-diabetic drugs are registered by the European Medicines Agency. We extracted available information regarding dose selection from public assessment reports and marketing application dossiers. Descriptive statistics were used to summarise the extracted information.

**Results:** In total, 14 drugs of three drug classes were included; sodium-glucose co-transporter-2 inhibitors (*n* = 4), dipeptidyl peptidase-4 inhibitors (*n* = 4) and glucagon-like peptide-1 receptor agonists (*n* = 6). For these drugs, 21 dose-finding trials were submitted including results of multiple off-target effects, of which body weight (*n* = 18) and low-density lipoprotein cholesterol (*n* = 14) were most frequently reported. Dose-response curves for off-target effects appeared to be different compared to the glycaemic dose-response curve. Glycated hemoglobin (100%) and fasting plasma glucose (42.9%), were used most frequently for the dose justification, but generally off-target effects (<25%) were not.

**Conclusions:** Dose justification to regulatory authorities was mainly based on glycaemic effects. The dose-response relationship for the off-target effects did not necessarily follow the dose-response relationship of the on-target effects suggesting that selection of the optimal anti-diabetic dose could benefit from including off-target effects in the dose selection process as well.

## Introduction

Type 2 diabetes is characterised by hyperglycaemia, which is associated with several symptoms, such as an increased frequency of urination, thirst and fatigue. In addition, patients with type 2 diabetes have an increased risk of micro- and macrovascular complications resulting in increased risk of cardiovascular morbidity and mortality (Davies et al., 2018; Committee for Medicinal Products for Human Use (CHMP), 2018). Therefore, management of patients is focused on improving quality of life and prevention or delay of complications associated with the disease (Davies et al., 2018; Committee for Medicinal Products for Human Use (CHMP), 2018). A fundamental aspect in the current treatment strategy is to optimise glycaemic control. Management of patients, however, should not be limited to optimising glycaemic control, but should also focus on addressing other cardiovascular risk markers. For instance, improved cardiovascular outcomes, independent of glycaemic control, are achieved by addressing other cardiovascular risk markers, such as lowering of systolic blood pressure (SBP) and improving lipid levels (Davies et al., 2018).

Results of cardiovascular outcome trials with new anti-diabetic therapies such as sodium-glucose co-transporter-2 (SGLT2) inhibitors and glucagon-like peptide-1 (GLP-1) receptor agonists demonstrate that these therapies decrease glycated hemoglobin (HbA1c), but also appear to confer cardiovascular and renal protection (Pfeffer et al., 2015; Zinman et al., 2015; Marso et al., 2016a; Marso et al., 2016b; Holman et al., 2017; Neal et al., 2017; Hernandez et al., 2018; Gerstein et al., 2019; Wiviott et al., 2019). There is controversy in the scientific literature, whether the renal and cardiovascular benefits of these novel anti-diabetic agents can be expected to be independent, or at least in part, of their effects to improve glycaemic control (Pfeffer et al., 2015; Zinman et al., 2015; Marso et al., 2016a; Marso et al., 2016b; Holman et al., 2017; Neal et al., 2017; Hernandez et al., 2018; Gerstein et al., 2019; Wiviott et al., 2019). Recent outcomes in non-diabetic populations with these drugs however appear to support that part of the renal and cardiovascular protection is independent of glycaemic control. Despite these additional cardiovascular and renal protective effects, the optimal dose of new anti-diabetic drugs is typically based solely on glycaemic risk markers and general tolerability considerations. For example, dose selection of SGLT2 inhibitors was based on the drug effects on urinary glucose excretion in combination with an overall safety assessment (Committee for Medicinal Products for Human Use (CHMP), 2012; Committee for Medicinal Products for Human Use (CHMP), 2013; Committee for Medicinal Products for Human Use (CHMP), 2014; Committee for Medicinal Products for Human Use (CHMP), 2018). SGLT2 inhibitors exert however, multiple effects, so called off-target effects, which may contribute to the long-term renal and cardiovascular benefits. These off-target drug effects are, however, often monitored less rigorously than the on-target drug effect and usually interpreted as safety effects (Heerspink et al., 2014).

Importantly, the dose-response relationship for off-target effects may be dissociated from the on-target effects as has been observed with renin-angiotensin-aldosterone system inhibitors where the blood pressure dose-response curve is different than the dose-response curve for albuminuria lowering (Heerspink and de Zeeuw, 2009). This raises the question whether the current dose selection procedure should be exclusively based on the on-target effects of a drug or if, in contrast, off-target effects should also be considered for dose selection.

From a regulatory perspective, the European Medicines Agency’s draft Guideline on clinical investigation of medicinal products in the treatment or prevention of diabetes mellitus (CPMP/EWP/1080/00 Rev. 2) recommends using fasting plasma glucose (FPG) as primary evaluation criterion in short-term dose-finding trials and HbA1c in dose-finding trials with a duration of more than 12 weeks (Committee for Medicinal Products for Human Use (CHMP), 2018). Justification of the selected dose range for phase 3 trials to regulatory authorities is therefore expected to focus mainly on glycaemic on-target effects, but it is currently unknown to what extent off-target effects are also considered in the dose justification of phase 3 trials to regulatory authorities.

This study aimed to evaluate which drug effects are currently included in the dose justification of phase 3 trials of drugs intended for the treatment of patients with type 2 diabetes. In addition, we evaluated whether the dose-response relationship of the off-target drug effects follows the dose-response relationship of the on-target glycaemic drug effects. Finally, we also evaluated whether there was any regulatory involvement that could have influenced the dose selection process.

## Materials and Methods

### Drugs Eligible for Inclusion

In the European Union, all drugs intended for the treatment or prevention of type 2 diabetes are registered centrally by the European Medicines Agency since 1995. Upon marketing application, a company submits a drug application dossier to the European Medicines Agency containing scientific evidence to support marketing approval. After review of the drug application dossier, the European Medicines Agency publishes full scientific assessment reports of authorised drugs, drugs refused from marketing authorisation or drugs suspended or withdrawn after approval, so called European Public Assessment Reports (EPAR). An EPAR summarises both the drug application dossier and the assessment of the marketing application dossier by regulatory authorities.

For this analysis, all anti-diabetic drugs with an EPAR and electronic drug application dossier were reviewed. The publicly available EPARs, were retrieved from the European Medicines Agency’s website (https://www.ema.europa.eu) up until December 2018. The non-publicly available drug application dossiers were accessed at the Dutch Medicines Evaluation Board. The focus of this analysis was on dose selection for drugs intended for the treatment of patients with type 2 diabetes, therefore only products with a registered indication for type 2 diabetes were included. Furthermore, fixed combination medicinal products (i.e. combination of two or more active substances), orphan drugs, insulins and generics were excluded.

### Review Process and Data Extraction

#### Evaluation of the dose justification of phase 3 trials to regulatory authorities

During marketing authorisation application, a clinical overview is presented by a company in the drug application dossier. This clinical overview is intended to provide a critical analysis of all available data submitted to support marketing authorisation, including a justification of the selected phase 3 dose (International Conference on Harmonisation of Technical Requirements for Registration of Pharmaceuticals for Human Use, 2016). For each drug, this justification was extracted to identify the primary dose-finding trials and to evaluate which drug effects were reported in the dose justification of the phase 3 trials. Subsequently, trial reports of the primary dose-finding trials were extracted from the drug application dossiers to evaluate which drug effects were investigated in these primary dose-finding trials. Extraction of drug effects focused on all pre-defined efficacy variables, which were considered to be drug effects of interest for dose selection. In addition, drug effects were categorised in on- and off-target effects, in which all glycaemic drug effects were considered to be on-target effects and all non-glycaemic drug effects were considered to be off-target effects. Furthermore, we also extracted information regarding patient population, statistical analyses and trial design used in the primary dose-finding trials to exclude major differences between trials.

#### Evaluation of the Dose-Response Relationship

After evaluating which drug effects were investigated in the dose-finding trials, we extracted, if available, their corresponding average effect size from the trial reports to graphically evaluate the dose-response relationship. Evaluation of the dose-response relationship focused on the most frequently reported on-target drug effects and off-target drug effects. In order to compare the dose-response relationships, we normalised the dose range by the maximum registered dose.

#### Evaluation of Regulatory Involvement in Dose Selection Process

Finally, we evaluated the regulatory involvement on the selection of the phase 3 dose range. In Europe, the European Medicines Agency provides voluntary scientific advice to drug developers to support the development of high-quality, effective and safe medicines in a timely manner (Regnstrom et al., 2010; Hofer et al., 2015). Selection of the phase 3 dose range could be influenced by advice received by the European Medicines Agency. Therefore, scientific advice procedures were identified from the initial marketing application forms, provided in the drug application dossier, and screened for questions related to selection of the phase 3 dose range. In addition, we evaluated regulatory involvement on dose selection during the review of the marketing application using the EPARs for any discussion around the phase 3 dose range at time of marketing authorisation application. Finally, we also evaluated whether there was agreement with the selected phase 3 dose by the regulatory authorities. Two independent reviewers screened all EPARs and extracted information regarding regulatory involvement at the time of marketing authorisation application. The results of the two reviewers were compared and checked for discrepancies. In case of a discrepancy, the results were based upon reaching consensus between the two reviewers.

### Data Presentation

Descriptive statistics were used to summarise the extracted information from the drug application dossiers and EPARs using R version 3.2.4 (R Foundation for Statistical Computing, Vienna, Austria). Graphical evaluation of the average dose-response relationship for all on- and off-target drug effects were also constructed in R using the ggplot2 package (version 3.0.0).

## Results

### Included Drugs

**FIGURE 1:**
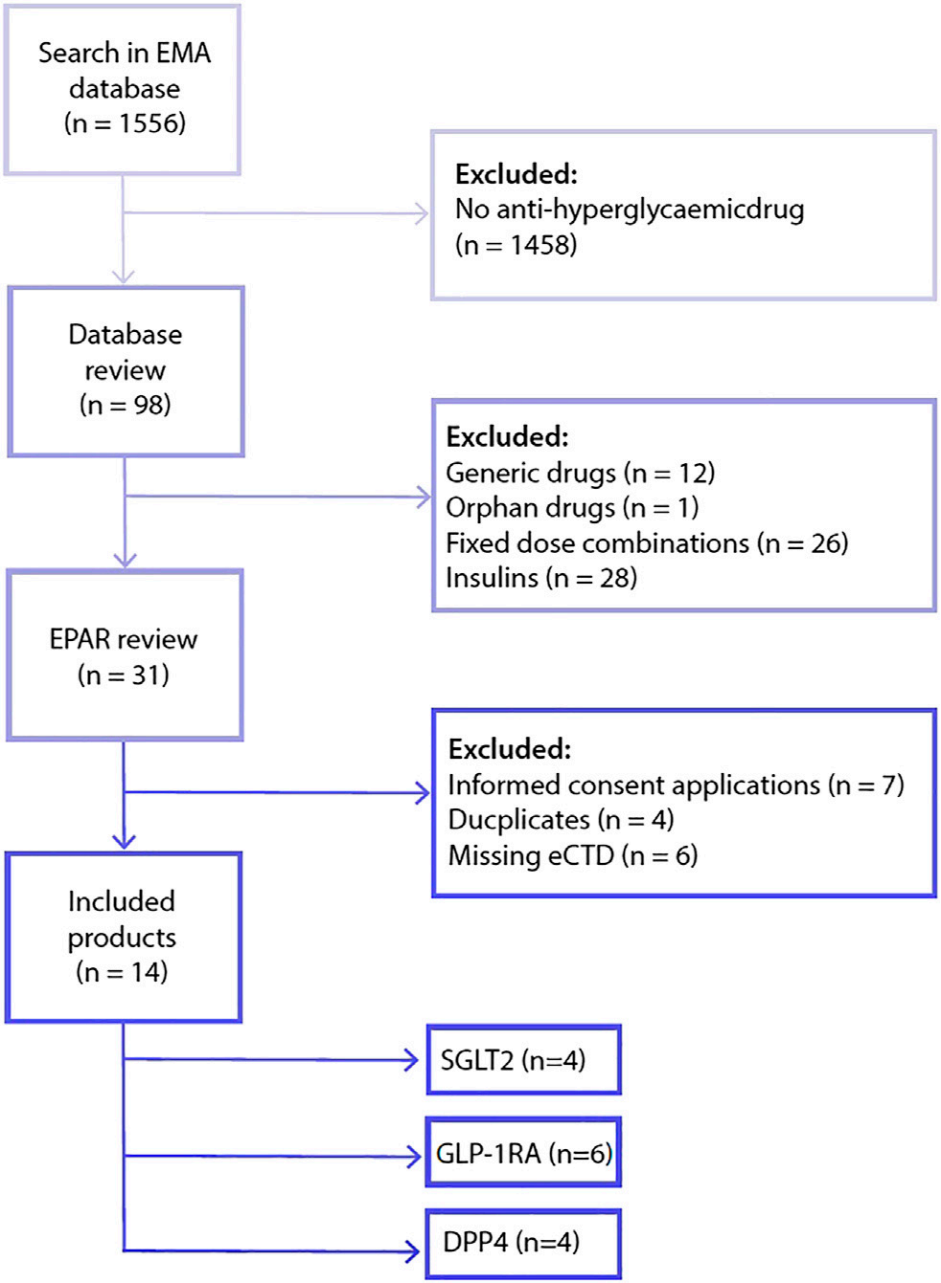
Flowchart included drugs. Abbreviations: dipeptidyl peptidase-4 (DPP4), electronic drug application dossier (eCTD), glucagon-like peptide-1 receptor agonist (GLP-1RA), sodium-glucose co-transporters -2 (SGLT2).

From 1995 to 2018, the European Medicines Agency received 98 marketing application procedures related to anti-diabetic drugs (Figure 1). After removal of generics, orphan drugs, fixed combination medicinal products and insulins, 31 drugs were eligible for inclusion in this study. From these 31 eligible agents, 17 were removed as these procedures were based on a duplicate drug application dossier (i.e. same applicant, same clinical dossier), an informed consent application (i.e. different applicant but same clinical dossier as the medicinal product referred to) or were missing an electronic drug application dossier. As a consequence, a total of 14 drugs were included in the analysis, comprising the three most recently registered drug classes; SGLT-2 inhibitors (*n* = 4 drugs), dipeptidyl peptidase-4 (DPP4) inhibitors (*n* = 4 drugs) and GLP-1 receptor agonists (*n* = 6 drugs).

### Evaluation of the Dose Justification of Phase 3 Trials to Regulatory Authorities

#### Trial Characteristics

A total of 21 primary dose-finding trials were used to justify the selected phase 3 dose. Characteristics of the included patient population, statistical analysis and design of these primary dose-finding trials are included in Table 1.

**TABLE 1:** Characteristics of the primary dose-finding trials used in justification in clinical overview.

			SGLT2 (*n* = 8 trials)	DPP4 (*n* = 6 trials)	GLP-1RA (*n* = 7 trials)	Total (*n* = 21 trials)
General characteristics	Drugs included in this analysis		4	4	6	14
	Total number of patients		2,923	2,536	1855	7,314
	Number of patients per trial		365	423	265	348
Patient population	Age	Years	56.6 (9.8)	56.1 (9.7)	55.2 (9.8)	56.1 (9.8)
	Gender	Female	1,211 (41.4)	1,152 (45.4)	821 (44.3)	3,184 (43.6)
		Male	1712 (58.6)	1,384 (54.6)	1,034 (55.7)	4,130 (56.4)
	Ethnicity	Afro-American	82 (2.8)	147 (5.8)	66 (3.6)	295 (4.0)
		Asian	1,072 (36.7)	53 (2.1)	301 (16.2)	1,426 (19.5)
		Caucasian	1,674 (57.2)	2076 (81.8)	1,186 (63.9)	4,936 (67.5)
		Other	95 (3.3)	260 (10.3)	302 (16.3)	657 (9.0)
	Body Mass Index	kg/m^2^	29.6 (5.2)	31.3 (4.9)	30.7 (5.0)	30.5 (5.1)
	Body weight	Kg	82.6 (18.7)	89.1 (17.2)	86.6 (17.4)	85.9 (18.1)
	HbA1c	%	7.9 (0.9)	8.0 (0.9)	8.0 (0.8)	7.9 (0.9)
	FPG	mg/dL	165.2 (41.7)	176.5 (43.3)	170.3 (43.6)	170.4 (42.3)
	Duration of diabetes	Years	5.0 (4.9)	4.6 (4.9)	5.1 (5.4)	5.0 (5.0)
	Co-morbidity					
		Nephropathy	3.8	1.8	1.7	2.4
		Neuropathy	6.8	8.2	7.7	7.5
		Retinopathy	4.1	4.0	2.6	3.5
Trial design	Number of dose levels		4	4	5	4
	Trial duration	Weeks	11	12	16	13
	Randomisation	Randomised (yes)	8	6	7	21
	Blinding	Double-blind (yes)	8	6	7	21
	Control					
		Active controlled	4	1	2	7
		Placebo controlled	8	6	7	21
	Clinical trial Design					
		Parallel design	8	6	7	21
		Adaptive design	0	0	1	1
	Phase in clinical development program					
		Phase 2	3	2	4	9
		Phase 2a	0	1	0	1
		Phase 2b	5	3	2	10
		Phase 2/3	0	0	1	1
	Background medication					
		Diet and exercise alone	3 (37.5)	1 (16.7)	0 (0.0)	4 (19.0)
		Metformin	4 (50.0)	1 (16.7)	3 (42.9)	8 (38.1)
		Maximum of 1 oral anti-diabetes drug	0 (0.0)	2 (33.3)	4 (57.1)	6 (28.6)
		Maximum of 2 oral anti-diabetes drug	1 (12.5)	2 (33.3)	0 (0.0)	3 (14.3)
Analyses	Statistical method					
		Descriptive statistics	8	6	7	21
		Conventional statistics (Analysis of Covariance)	8	6	6	20
		Dose-Response (non-linear regression)	2	0	1	3
		Population Pharmacokinetic or Pharmacokinetic/Pharmacodynamic analysis	2	2	3	7
	Imputation method					
		Last Observation Carried Forward	8	6	7	21
	Pharmacokinetics					
		Plasma-concentrations measured	8	5	7	20
	Type of samples taken					
		Trough concentrations	3	3	1	7
		Sparse	5	2	5	12
		Dense	0	0	1	1

Data are presented as pooled mean (SD) and number of trials/percentage of total number of trials.

Abbreviations dipeptidyl peptidase-4 (DPP4), fasting plasma glucose (FPG), glucagon-like peptide-1 receptor agonist (GLP-1RA), glycated hemoglobin (HbA1c), sodium-glucose co-transporters -2 (SGLT2).

In short, all dose-finding trials were randomised, double-blind, parallel-design and placebo-controlled clinical trials with a mean duration of 13 weeks. Four dose levels were typically included per dose-finding trial. Patients had a mean age of 56.1 years (±9.8 years), were predominantly Caucasian (67.5%), male (56.4%) and had a mean body mass index of 30.5 kg/m^2^ (±5.1 kg/m^2^). Furthermore, patients had a mean duration of disease of 5.0 years (±5.0 years), baseline HbA1c of 7.9% (±0.9%) and a FPG of 170.4 mg/dl (±42.3 mg/dl). No major differences in patient populations were present between dose-finding trials of the different drug classes, except for the SGLT2 inhibitors, where more patients of Asian descent were included compared to the other two classes.

#### Drug Effects Included in Dose-Finding Trials

**FIGURE 2:**
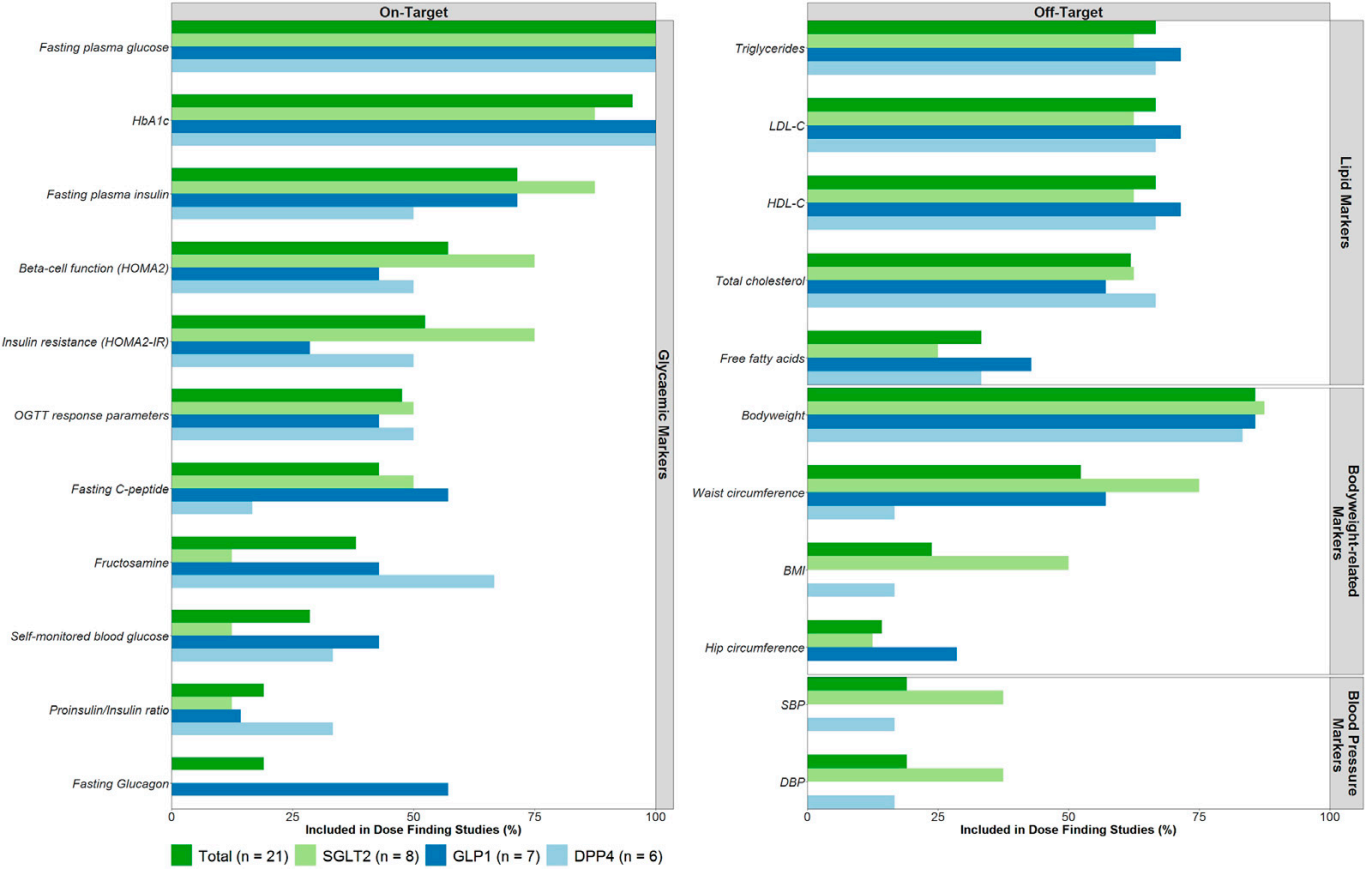
On- and Off-target effects included as efficacy endpoints in dose-finding trials. Abbreviations: Body Mass Index (BMI), Diastolic blood pressure (DBP), dipeptidyl peptidase-4 (DPP4), glucagon-like peptide-1 (GLP1), glycated hemoglobin (HbA1c), high-density lipoprotein cholesterol (HDL-C), low-density lipoprotein cholesterol (LDL-C), sodium-glucose co-transporters -2 (SGLT2), systolic blood pressure (SBP).

The on-target drug effects, FPG (100%) and HbA1c (95.2%), were most frequently investigated in the dose-finding trials (Figure 2). Off-target drug effects measured in the dose-finding trials primarily focused on lipid markers, body weight-related markers and blood pressure-related markers. Of these off-target drug effects, body weight was reported most frequently (85.7%) followed by triglycerides (66.7%), low-density lipoprotein cholesterol (LDL-C, 66.7%), high-density lipoprotein cholesterol (HDL-C, 66.7%) and total cholesterol (61.9%). No major differences between drug classes were noted in the number of included on- and off-target effects measured.

#### Drug Effects Included in Dose Justification of Phase 3 Trials

**FIGURE 3:**
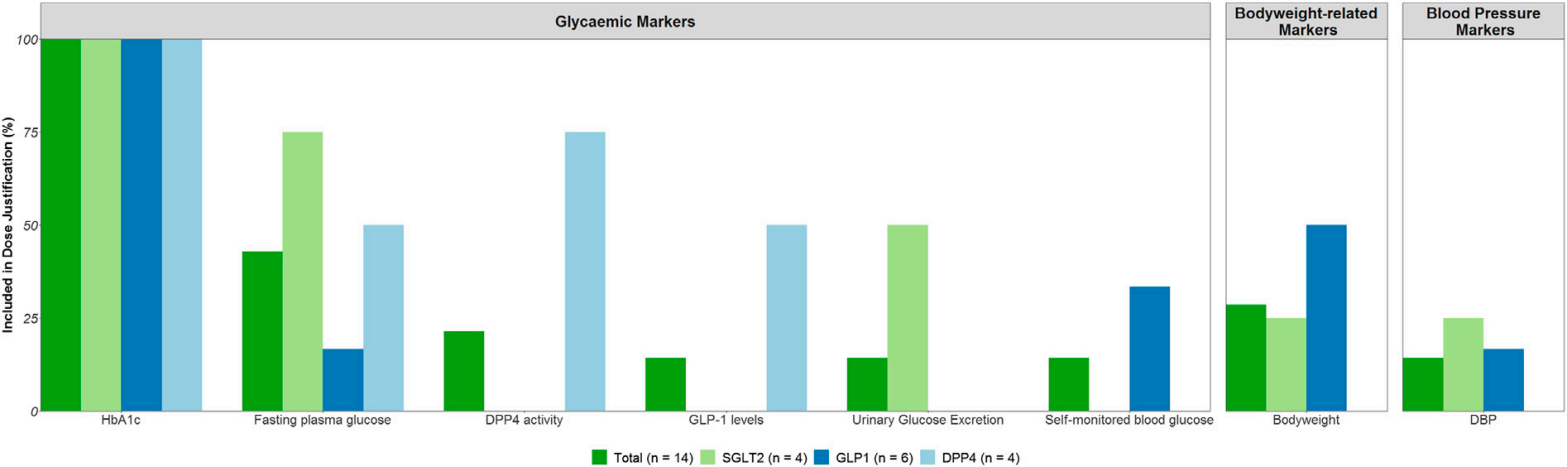
On- and Off-target effects included in the justification of the phase 3 dose range. Abbreviations: Diastolic blood pressure (DBP), dipeptidyl peptidase-4 (DPP4), fasting plasma glucose (FPG), glucagon-like peptide-1 (GLP1), glycated hemoglobin (HbA1c), sodium-glucose co-transporters -2 (SGLT2).

A total of eight different risk markers were included in the dose justification of phase 3 trials for more than one drug (Figure 3).

For the on-target drug effects, primarily HbA1c (*n* = 14) was used in the dose justification for all drugs, followed by FPG (*n* = 6). For two out of the four DPP4 inhibitors, DPP4 activity and GLP-1 levels were also included in the dose justification in addition to HbA1c and FPG. Further, for two out of the four SGLT2 inhibitors, urinary glucose excretion was included in the dose justification next to HbA1c and FPG.

For the off-target effects, body weight (*n* = 4) and diastolic blood pressure (DBP, *n* = 2) were reported in the dose justification. These effects were reported for the GLP-1 receptor agonists and the SGLT2 inhibitors, but a majority of drugs did not consider any off-target effects in the dose justification.

### Evaluation of the Dose-Response Relationship

**FIGURE 4:**
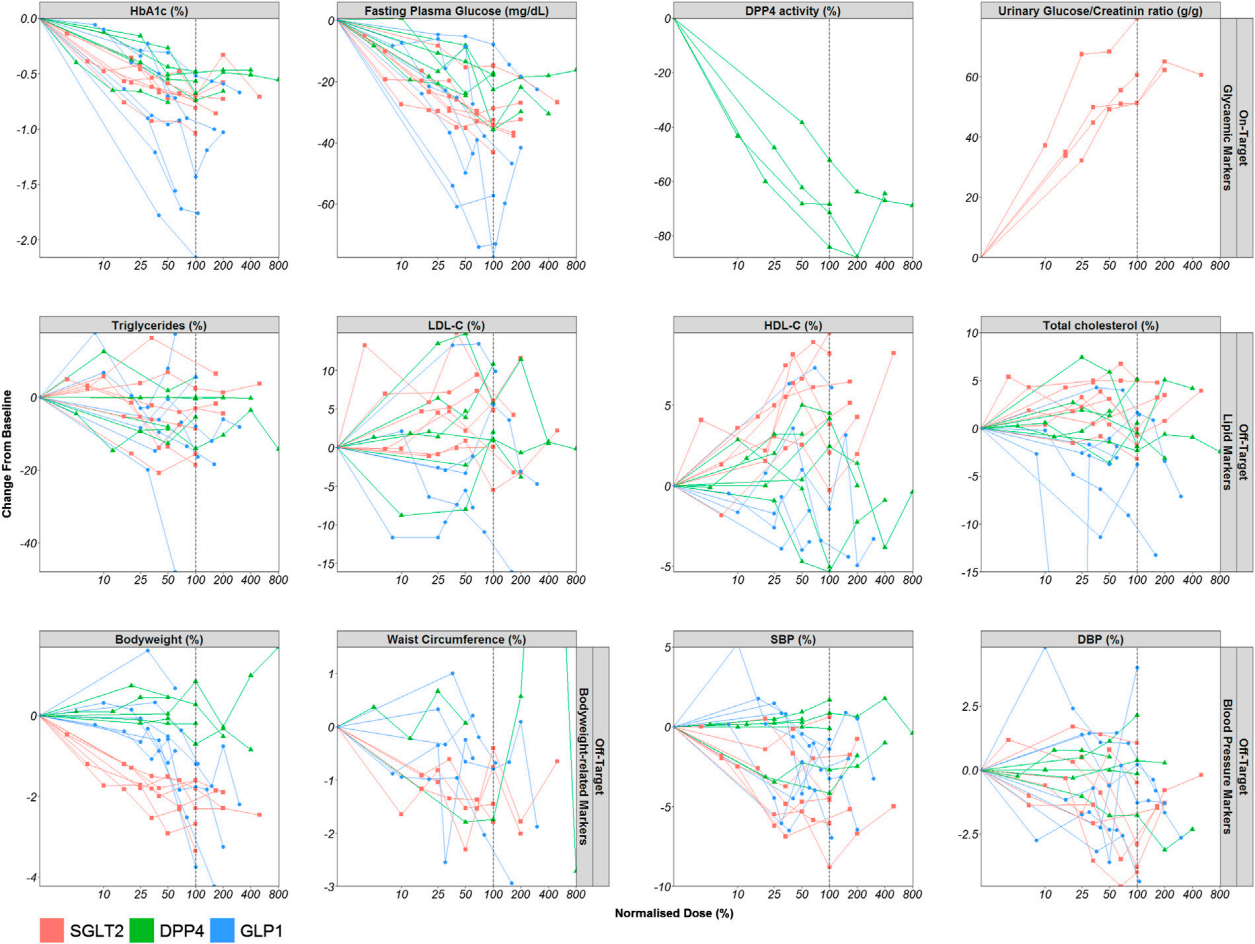
Dose-Response relationship of markers of the on- and off-target drug effects. Mean observed dose-normalised drug effect (o) per dose level are displayed for all included dose-finding trials (lines). Abbreviations: Diastolic blood pressure (DBP), dipeptidyl peptidase-4 (DPP4), fasting plasma glucose (FPG), glucagon-like peptide-1 (GLP1), glycated hemoglobin (HbA1c), high-density lipoprotein cholesterol (HDL-C), low-density lipoprotein cholesterol (LDL-C), sodium-glucose co-transporters -2 (SGLT2), systolic blood pressure (SBP).

The dose-response relationships for the most frequently reported on-target and off-target effects stratified by drug class are displayed in Figure 4. Figures per drug effect and per drug class are provided in the Supplemental materials. Every line represents the dose-response relationship observed per dose-finding trial. The effects observed in the on-target and off-target effects are normalised by the highest registered dose (e.g. 10 mg dapagliflozin reflects the 100% dose level).

For all drug classes, upon visual inspection, clear dose-response relationships were observed for HbA1c and FPG. In addition, the effects of DPP4 inhibitors on DPP4 activity also showed a dose-dependent effect and the same held true for the effect of SGLT2 inhibitors on urinary glucose to creatinine ratio. SGLT2 inhibitors and GLP1 receptor agonists also displayed a dose-dependent effect on body weight, HDL-C and SBP.

For SGLT2 inhibitors, the dose-response relationship of body weight, HDL-C and SBP appears to follow a similar relationship as HbA1c with dose, although it is not completely clear whether maximum effects in HDL-C and SBP have already been reached with the evaluated dose levels. For GLP1 receptor agonists, the dose-response relationship of body weight and SBP seems to be shifted to the right compared to the dose-response relationship for HbA1c so that the effect became apparent only at higher doses. For DPP4 inhibitors, there are no clear dose-response relationships for the off-target parameters. In general, for all drug classes, the dose-response relationship of the other off-target effects appears to be more variable.

### Evaluation of Regulatory Involvement in the Dose Selection Process

Regulatory involvement in the dose selection process is displayed in Table 2. The dosages included in the primary dose-finding trials are mainly based on the results of the earlier single- and multiple-ascending phase one dose trials. In general, a wide range of dosages is included in the primary dose-finding trials. Typically two dosages were proposed for the phase 3 trials and, in most cases, both dosages were also registered.

**TABLE 2:** Review of regulatory process.

	Primary dose-finding trials		Justification company		Regulatory involvement			
Drug name	Selected dose range	Justification dose range	Proposed phase 3 dosages	Primary efficacy variable	Scientific advice regarding phase 3 dose	Phase 3 dose range discussed in EPAR	Agreement in EPAR with phase 3 dose	Registered dosages
SGLT2-inhibitors								
Dapagliflozin	2.5–50 mg per day	Results of single and multiple ascending dose trials	1.0, 2.5, 5.0, 10.0 mg	HbA1c	No	Yes	Yes	5 and 10 mg per day
Ertugliflozin	1.0–25 mg per day	Results of single and multiple ascending dose trials, dose-response modeling	5 mg, 15 mg	HbA1c, SBP	Yes	Yes	Yes	5 and 15 mg per day
Canagliflozin	50.0–600.0 mg per day	Results of multiple ascending dose trial	100 mg, 300 mg	HbA1c	No	Yes	Yes	100 and 300 mg per day
Empagliflozin	1.0–50.0 mg per day	Results of single and multiple ascending dose trials	10 mg, 25 mg	HbA1c	No	Yes	Yes	10 and 25 mg per day
*DPP4-inhibitors*								
Sitagliptin	10.0–100.0 mg per day	Results of single ascending dose trial, concentration-response modeling	100 mg, 200 mg	HbA1c	Yes	Yes	Yes, but 200 mg dose not mentioned in EPAR	25, 50 and 100 mg per day
Alogliptin	6.25–100.0 mg per day	Results of multiple ascending dose trial	12.5 mg, 25.0 mg	HbA1c	Yes	Yes	Yes	6.25, 12.5 and 25.0 mg per day
Saxagliptin	2.5–40.0 mg per day	Results of single and multiple ascending dose trials	2.5, 5.0, 10.0 mg	HbA1c	Yes	Yes	No, the regulatory authorities were uncertain whether 5.0 mg was the most optimal dose. No difference in response between 2.5 and 5.0 mg was observed	2.5 and 5.0 mg per day
Linagliptin	1.0–10.0 mg per day	Results of multiple ascending dose trial	5 mg	HbA1c	No	Yes	Yes	5.0 mg per day
*GLP-1* receptor agonists								
Semaglutide	0.1–1.6 mg per week	Results of single ascending dose trial, concentration-response modeling	0.25, 0.5, 1.0 mg	HbA1c	Yes	Yes	Yes, regulatory authorities concluded that there was no unequivocal evidence for a better benefit/risk profile with semaglutide 0.5 and 1.0 mg in the phase 3 trials compared to 0.4 and 0.8 mg semaglutide. However, regulatory authorities accepted the 0.5 and 1.0 mg as these dosages were included in clinical phase 3 trials	0.25, 0.5 and 1.0 mg per week
Dulaglutide	0.25–3.0 mg per week	Results of single and multiple ascending dose trials, dose-concentration-response modeling	0.75 mg, 1.5 mg	HbA1c	Yes	Yes	No, during assessment, regulatory authoritiesrequested to make the 0.75 mg strength formulation available, to which the applicant agreed. Reason for this was that for more vulnerable groups, a starting dose of 0.75 mg would provide more optimal benefit/risk	0.75 and 1.5 mg per week
Exenatide	0.8–2.0 mg per week	Dose-concentration modeling	0.8 mg, 2.0 mg	HbA1c	Yes	Yes	Not reported	2 mg per week
Liraglutide	0.65–1.9 mg per day	Results of multiple ascending dose trial	0.6, 1.2, 1.8 mg	HbA1c	No	Yes	Yes, but regulatory authorities concluded that a clear justification for the 1.2 and 1.8 mg dosages was lacking as mainly lower dosages were used in the pharmacodynamic trials. Nonetheless, efficacy was demonstrated for these dosages and were therefore accepted	0.6, 1.2 and 1.8 mg per day
Albiglutide	4.0–30.0 mg per week, 15.0–50.0 mg biweekly and 100.0 mg every four weeks	Results of multiple ascending dose trial, concentration-response modeling	30 mg, 50 mg	HbA1c	No	Yes	Yes	30 and 50 mg per week
Lixisenatide	5.0–30.0 µg per day	Previous toxicity and previous clinical trials	10 μg, 20 µg	HbA1c	No	Yes	Yes	10 and 20 µg per day

Abbreviations dipeptidyl peptidase-4 (DPP4), European Public Assessment Report (EPAR), glucagon-like peptide-1 (GLP-1), glycated hemoglobin (HbA1c), sodium-glucose co-transporters -2 (SGLT2), systolic blood pressure (SBP).

Scientific advice was requested for almost all drugs before submission of the marketing authorisation application, but the scientific advice was only for seven products related to the phase 3 dose. The justification of the phase 3 dose has been discussed in all EPARs, except for one drug. For nine drugs, regulatory authorities agreed with the selected dose without expressing uncertainties. For two drugs there was disagreement on the selected dose, and for one of these drugs this resulted in additional registration of a lower dose than originally applied for. Further, for one drug, no agreement or disagreement was reported. For another two drugs, uncertainty with respect to the selected dose was expressed by the regulatory authorities.

## Discussion

In this study, we evaluated the dose justification of phase 3 trials of SGLT-2 inhibitors, DPP4 inhibitors and GLP-1 receptor agonists, three of the most recently registered anti-diabetic drug classes. The dose of these drug classes was determined based on results of several primary dose-finding trials, which typically investigated the on-target drug effects, FPG and HbA1c. In these dose-finding trials, off-target drug effects were however also frequently measured, which were predominantly related to body weight, lipid profile and blood pressure. Even though dose-finding trials measured off-target effects, the dose justification of phase 3 trials to regulatory agencies was, for a majority of drugs, solely based on the on-target effects. The dose-response relationship for the off-target effects did not necessarily follow the dose-response relationship of the on-target effects suggesting that selection of the optimal anti-diabetic dose could benefit from including off-target effects in the dose selection process as well.

The new draft Guideline on clinical investigation of medicinal products in the treatment or prevention of diabetes mellitus (CPMP/EWP/1080/00 Rev. 2) recommends using FPG and HbA1c as primary evaluation criterion in dose-finding trials (Committee for Medicinal Products for Human Use (CHMP), 2018). For the assessment of efficacy trials, the Guideline states that it is also important to consider other cardiovascular risk markers (Committee for Medicinal Products for Human Use (CHMP), 2018). In particular, serum lipids, body weight or body composition as well as blood pressure and heart rate are explicitly mentioned (Committee for Medicinal Products for Human Use (CHMP), 2018). In line with the Guideline, these off-target drug effects were also most frequently investigated in dose-finding trials included in our analysis. They were, however, not considered explicitly in the dose justification of phase 3 trials to the regulatory authorities. This raises the question whether regulators should request evaluation of both on-target and off-target effects for the dose justification of phase 3 trials. Especially since cardiovascular and renal outcomes are influenced by multiple risk factors, not only glycaemic control, and anti-diabetic treatments have been shown to influence multiple cardiovascular risk markers (both positively and negatively).

We defined all glycaemic drug effects as on-target effects and non-glycaemic drug effects as off-target effects to align with the draft Guideline on clinical investigation of medicinal products in the treatment or prevention of diabetes mellitus (CPMP/EWP/1080/00 Rev. 2), which makes the distinction between glycaemic effects and other cardiovascular effects. It can be argued that changes in off-target effects are direct consequences of improvements in on-target effects. It can however not be excluded that other mechanisms, with potentially a different relationship between dose and response, also contribute to changes in the off-target effects of new anti-diabetic drugs.

It is acknowledged that assessment and inclusion of off-target drug effects in the dose decision making process is redundant when the dose-response relationship for the on-target drug effects is similar to the off-target dose-response relationship. No additional benefit can then be expected to be gained for including off-target effects in dose selection. It appears however that, for at least the GLP-1 receptor agonists and potentially also for SGLT-2 inhibitors, differences exist between the on-target and off-target dose-response relationship. For example, for GLP-1 receptor agonists approximately maximum effects on HbA1c are reached with the highest registered therapeutic dose, but this is not the case for the off-target drug effect on body weight. This ultimately has led to higher dose levels being approved for GLP-1 receptor agonists in the management of body weight after these products demonstrated adequate long-term efficacy and safety for this indication. Additionally, for the SGLT-2 inhibitor dapagliflozin, it was recently suggested that the dose-response relationship varies between glycaemic and non-glycaemic risk markers (Koomen et al., 2020). Thus, selecting a dose for phase 3 trials solely based on the on-target relationship seems inadequate to determine an optimal dose for cardiovascular and renal protection of patients with type 2 diabetes mellitus. Additional benefit for patients may be achieved by optimising the dose on a panel of on-target and off-target drug effects. Similar approaches have been described in the literature, in which short term changes in several on- and off-target drug effects are integrated to predict the long term cardiovascular or renal outcome (Smink et al., 2014a; Smink et al., 2014b; Schievink et al., 2016).

For one drug included in the analysis, dose selection was based on a panel of drug effects in the initial phase of the trial and subsequently confirmed in a long-term period (Skrivanek et al., 2014). The dose of dulaglutide was selected in a phase 2/3 trial, in which a Bayesian algorithm was used based on two efficacy measures, HbA1c and body weight, and two safety measures, pulse rate and diastolic blood pressure (Skrivanek et al., 2014). For each of these measures, pre-defined criteria were determined and incorporated in a clinical utility index to facilitate dose selection (Skrivanek et al., 2014). This resulted in selection of the 0.75 and 1.5 mg dosages for the long-term confirmatory period of the trial (Skrivanek et al., 2014). Advantages of this approach are the ability to include prior information in the dose selection process and also the ability to objectively quantify the benefit-risk profile in the dose selection process (Skrivanek et al., 2014). However, a major limitation to this approach, also raised by the investigators, is that other drug effects than those included in the algorithm, e.g., because they were not measured, could also be important for dose selection (Skrivanek et al., 2014). It is currently unclear which markers and how many markers would be necessary to determine the optimal dose for a new anti-diabetic therapy. Future research focusing on potential mechanisms for cardiovascular protection and harm will be essential to facilitate dose selection of new drugs using similar approaches. Nonetheless, we believe the approach used for the dose selection of dulaglutide is a step in the right direction as it reflects both on-target as well as the off-target drug effects of dulaglutide. The benefit/risk balance is therefore not only evaluated in the confirmatory part of the drug development program, but also in an earlier stage during dose selection.

Scientific advice related to the selection of the phase 3 dose was received in half of the drugs included in this analysis, which indicates that there could be a significant regulatory involvement in the dose selection process before marketing authorisation application. Nonetheless, for two drugs that received scientific advice, the regulatory authorities disagreed with the dose range applied for at the time of marketing authorisation application. Yet, only for dulaglutide, the regulatory authorities requested to make, in addition to the 1.5 mg strength applied for, the 0.75 mg strength available for more vulnerable patient groups as a starting dose. In general, it therefore appears that the regulatory authorities may have had a limited impact on the dose selection process.

This study has some limitations. First of all, we extracted all drug effects investigated in dose-finding trials that were reported as efficacy variables. Dose-finding trials are usually not powered to detect specific safety events and usually no specific safety events are defined in dose-finding trials. Therefore, the influence of safety on the dose selection process has not been taken into account in this analysis. Second, the trial duration of the dose-finding trials was not always comparable. Therefore, the effect size of the drug effects cannot be directly compared between drugs. Nonetheless, the trends in the normalised dose-response relationships can be compared as these are based on individual trial results, which are not influenced by external factors. Third, this analysis is based on short-term changes in on-target and off-target drug effects. Therefore, even though positive effects are observed in several off-target drug effects with non-registered dosages, these findings will need to be confirmed in long-term phase 3 trials. Finally, the assessment of regulatory involvement was limited to public available assessment reports due to data protection policies. Therefore, the regulatory influence on dose selection could have been underestimated.

In conclusion, for drugs intended for the treatment or prevention of type 2 diabetes mellitus, justification of the phase 3 dose was mainly based on the on-target drug effects and not on the off-target drug effects. Nonetheless, multiple off-target drug effects, such as lipids, blood pressure and body weight are measured in dose-finding trials. The dose-response relationship of the off-target effects does not necessarily follow the on-target drug effects. Since renal and cardiovascular risk is determined by multiple risk markers dose selection could benefit from including off-target risk markers in the dose selection process, in addition to the on-target effects of a drug, in case the dose-response relationship is different between risk markers. An integrated approach accounting for the on-target and off-target effects is necessary to select and justify the optimal dose for phase 3 trials and potentially marketing authorisation.

## Data Availability

The data contained in this article and as presented in the paper can be shared. These data are available upon request in the form of aggregated data per drug class or as partly de-identified data for individual drugs.

## Abbreviations

BMI, Body Mass Index; DBP, Diastolic Blood Pressure; DPP4, Dipeptidyl Peptidase-4; eCTD, Electronic drug application dossier, EPAR, European Public Assessment Reports; FPG, Fasting Plasma Glucose; GLP1, Glucagon-Like Peptide one; HbA1c, Glycated hemoglobin; HDL-C, High-density Lipoprotein Cholesterol; LDL-C, Low-density Lipoprotein Cholesterol; SBP, Systolic Blood Pressure; SGLT2, Sodium-Glucose co-transporter-2.
