# Supplementary material for: A Review of the Dose Justification of Phase 3 Trials to Regulatory Authorities for Drugs Intended for the Treatment of Type 2 Diabetes in Europe
Source: Front Pharmacol. 2021 Apr 28;12:626766. doi: 10.3389/fphar.2021.626766 (PMC8113630; doi:10.3389/fphar.2021.626766)
Supplement: Supplementary file 1 [file image1.pdf]

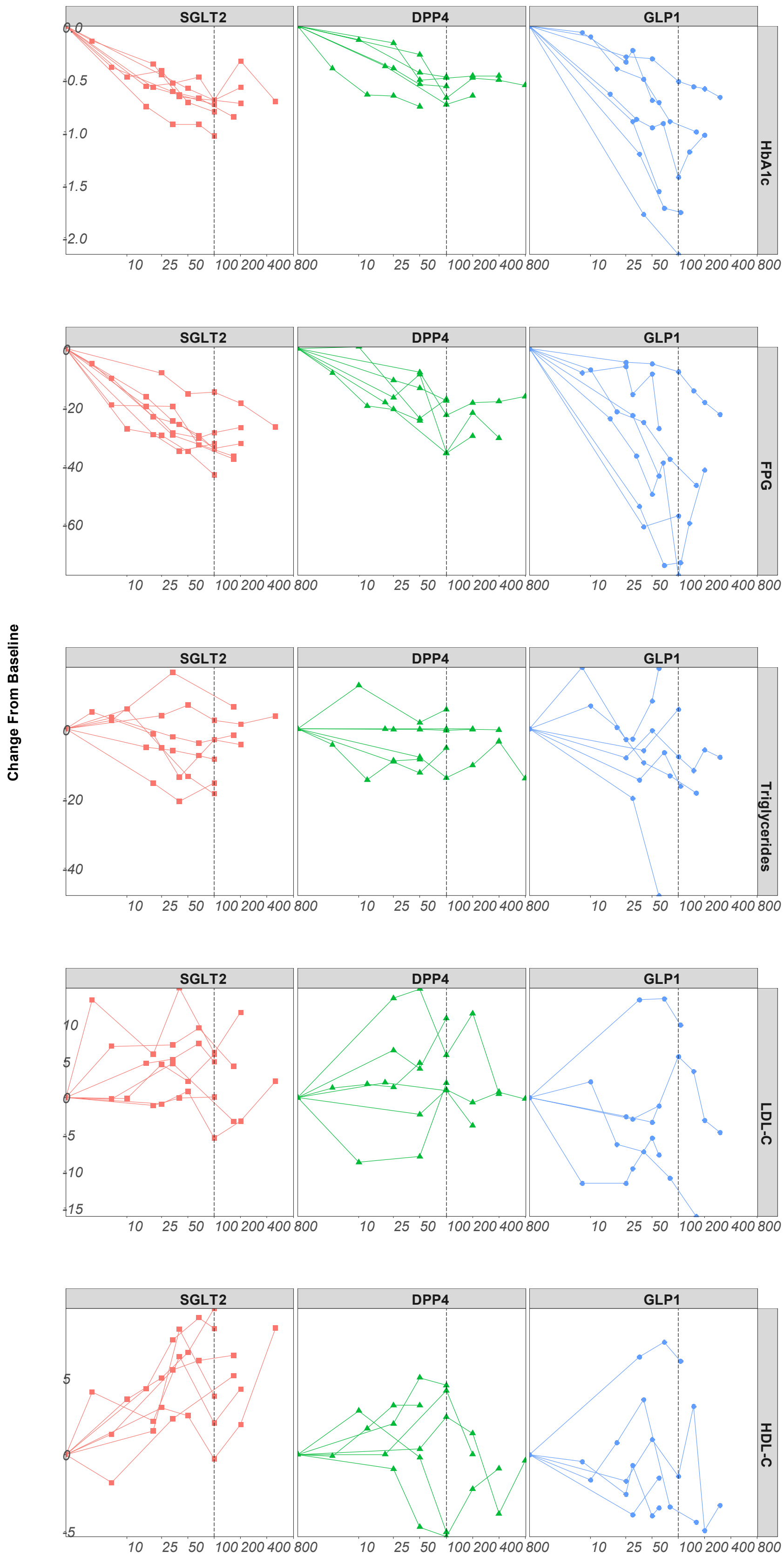
Change From Baseline
SGLT2
DPP4
GLP1
HbA1c
FPG
Triglycerides
LDL-C
HDL-C
0.0
-0.5
-1.0
-1.5
-2.0
0
-20
-40
-60
10
5
-5
-10
-15
10 25 50 100 200 400 800

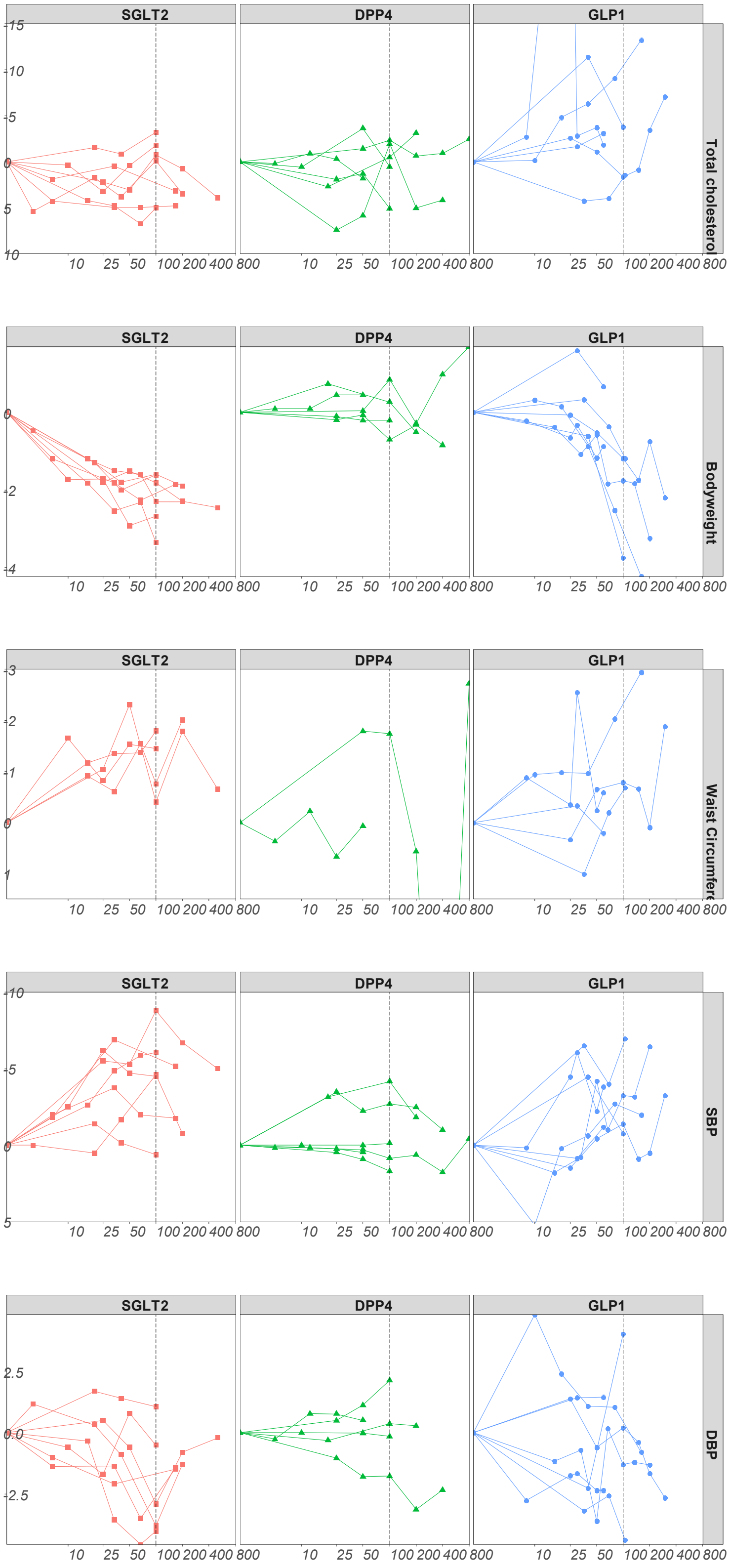

SGLT2
DPP4
GLP1
Total cholesterol
Bodyweight
Waist Circumfer
SBP
DBP
10 25 50 100 200 400 800

Figure S1. Dose-Response relationship of markers of the on- and off-target drug effects stratified by marker. Mean observed dose-normalised drug effect per dose level are displayed for all included dose-finding trials (lines). Abbreviations: Diastolic blood pressure (DBP), dipeptidyl peptidase-4 (DPP4), fasting plasma glucose (FPG), glucagon-like peptide-1 (GLP1), glycated haemoglobin (HbA1c), high-density lipoprotein cholesterol (HDL-C), low-density lipoprotein cholesterol (LDL-C), sodium-glucose co-transporters -2 (SGLT2), systolic blood pressure (SBP).
